# Poly(vinylidene Fluoride-Hexafluoropropylene) Porous Membrane with Controllable Structure and Applications in Efficient Oil/Water Separation

**DOI:** 10.3390/ma11030443

**Published:** 2018-03-18

**Authors:** Xinya Wang, Changfa Xiao, Hailiang Liu, Qinglin Huang, Junqiang Hao, Hao Fu

**Affiliations:** State Key Laboratory of Separation Membranes and Membrane Processes, National Center for International Joint Research on Separation Membranes, Tianjin Polytechnic University, No. 399, Binshui Road, Xiqing District, Tianjin 300387, China; wangxy0914@163.com (X.W.); liuhailiang723@163.com (H.L.); huangqinglin@tjpu.edu.cn (Q.H.); 15222696965@163.com (J.H.); fuhao0823@163.com (H.F.)

**Keywords:** PVDF-HFP, thermally induced phase separation, mixed diluent, hybrid membrane, water-in-oil emulsion separation

## Abstract

Poly(vinylidene fluoride-hexafluoropropylene) (PVDF-HFP) porous membranes are fabricated via thermally induced phase separation (TIPS) with mixed diluent (dibutyl phthalate (DBP)/dioctyl phthalate (DOP)). The effects of mixed diluent are discussed in detail in term of morphology, mean pore size, selective wettability, etc. The results show that the membrane structure changes from spherulitic to bicontinuous with the change of DBP/DOP ratio. It is also found that the degree of crystallization decreases with the decrease of DBP/DOP ratio in mixed diluent. When liquid–liquid (L-L) phase separation precedes solid–liquid (S-L) phase separation, the obtained membranes have outstanding hydrophobicity and lipophilicity, excellent mechanical property. Additionally, the PVDF-HFP hybrid membranes are prepared with silica (SiO_2_) particles and the effect of SiO_2_ content on structure and properties is discussed. It is found that the PVDF-HFP hybrid membrane with 2 wt % SiO_2_ (M3-S2) has better properties and higher filtration rate and separation efficiency for surfactant-stabilized water-in-oil emulsion separation. Moreover, the membrane M3-S2 also exhibits excellent antifouling performance for long-running.

## 1. Introduction

**Figure 1:**
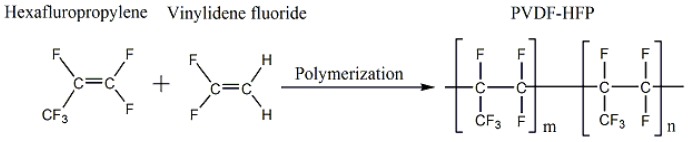
Polymerization equation of PVDF-HFP.

Recently, an emerging fluoro-copolymer, PVDF-HFP, is attracting more and more attention. As shown in Figure 1, PVDF-HFP can be obtained via emulsion polymerization of vinylidene fluoride (VDF) and hexafluoropropylene (HFP) [1]. Compared with polyvinylidene fluoride (PVDF), PVDF-HFP has better properties, such as higher solubility, higher hydrophobicity and better mechanical strength, owing to the combination of HFP [2,3,4]. To date, most studies focused on the fabrication of PVDF-HFP membranes via non-solvent induced phase separation (NIPS) method and the obtained membranes were applied in processes of membrane distillation (MD), polymer battery and gas absorption, etc. Khayet et al. [5] prepared PVDF-HFP hollow fiber membranes via NIPS method and studied the effects of polymer concentrations on MD process. Hemmat et al. [6] studied the effects of three salt additives (CaCO_3_, LiCl, and CaCl_2_) on MD performance of PVDF-HFP membrane. Wang et al. [7] prepared PVDF-HFP flat-sheet asymmetric membranes and investigated the effects of molecular weight of PEG on performance for MD process. Khayet et al. [8] investigated the effects of solvent compositions (*N*,*N*-dimethylformamide (DMF), DMAC and trimethylphosphate (TMP)) on the PVDF-HFP hollow fiber membrane structure and MD performance. Stephan et al. [9], Hwang et al. [10] and Li et al. [11] prepared PVDF-HFP flat-sheet membranes and applied them in polymer battery technology. Additionally, Jiraratananon et al. [12] fabricated modified PVDF-HFP hollow fiber membrane by using the method of dehydrofluorination and applied it in a membrane gas absorption process. However, there are very few studies on fabrication of PVDF-HFP membrane via TIPS method. Cui et al. [13] prepared PVDF-HFP membranes with sulfolane via TIPS method for lithium ion batteries. Cao et al. [14] investigated the CO_2_ extraction of DBP from PVDF-HFP/DBP former films prepared via TIPS.

During TIPS process, the diluent is a key factor for controlling the membrane structure. The diluent compositions influenced the interaction between the diluent and polymer which have a significant effect on membrane structure [15]. Li et al. [16], Song et al. [17] and Wang et al. [18] prepared PVDF flat/hollow fiber membranes with mixed diluents via TIPS method and studied the effects of diluent compositions on membrane structure and performance. It was found that desirable structure and outstanding performance can be obtained by changing diluent compositions. Nevertheless, the membrane prepared via TIPS method shows unsatisfactory properties. Thus, the modification method is becoming more important for membrane properties. Sun et al. [19] fabricated PVDF hollow fibers and flat sheet membranes with hydrophilic surface via amine treatment. Zhang et al. [20] modified PVDF membrane surface by doping ammonia water into polymer solution and formed micro/nanostructure via the modified phase-inversion process. Wang et al. [21] attempted to fabricate PVDF membranes modified with Au nanoparticles which provided sufficient capacity for continuous reduction of p-nitrophenol (4-NP) and methylene blue (MB) in chloroform-in-water emulsion. Hou et al. [22] investigated the preparation of polytetrafluoroethylene (PTFE) composite membrane with a hydrophilic poly(vinyl alcohol)/silica nanoparticles (PVA-Si) hybrid fibrous coating and the obtained membrane has a hydrophobic substrate and an in-air hydrophilic and underwater superoleophobic top surface. Graphene membranes covalently modified with polydimethylsiloxane were prepared by Lei et al. [23] and presented the potential for water/oil separation. However, these methods are difficult to scale up owing low maneuverability and poor stability. Compared with various modification methods, direct blending inorganic materials with polymers is easy and effective. 

In this study, PVDF-HFP and mixed diluent (DBP)/(DOP) are used for fabricating porous membranes via TIPS method. The effect of the interaction between mixed diluent and PVDF-HFP on the structure of obtained membranes is investigated and the structure is controlled through changing the composition of mixed diluent. Additionally, PVDF-HFP hybrid membranes are fabricated by blending hydrophobic SiO_2_ particles and the effects of SiO_2_ contents on structure and performance are discussed in detail.

## 2. Experimental

### 2.1. Materials

PVDF-HFP (Kynar2500, ≈18 mol % HFP), semi-crystalline copolymer, was purchased from Arkema (Singapore). DBP (b.p. 340 °C) and DOP (b.p. 386 °C) were purchased from Tianjin Kermel Chemical Reagent Co. Ltd. (Tianjin, China) Silica particles (SiO_2_ average particle size 40 nm were purchased from Guangzhou GBS High-tech & Industry Co., Ltd. (Guangzhou, China) Graphene (KNG-G5) was purchased from Xiamen Knano Graphene Technology Co., Ltd. (Xiamen, China) Alcohol, used as an extractant, was provided by AR grade. Diesel was purchased from Tianjin Fengchuan Chemical Reagent Technologies Co. Ltd. (Tianjin, China).

### 2.2. Determination of Phase Diagram

Mixed diluent (DBP and DOP) was mixed with 30 wt % PVDF-HFP at an appropriate temperature (150 °C) in a clean glass vessel and stirred for approximately 3 h to get homogeneous solution. Then, the glass vessel was directly quenched in liquid nitrogen to obtain solid polymer–diluent mixtures. To measure cloud points, a small amount of solid polymer–diluent mixtures was heated to 180 °C on a universal heat-cool stage (THMS 600, Linkam, Surrey, UK) using 20 °C·min^−1^ heating rate and held for 1 min, then cooled to room temperature using the cooling rate of 5 °C·min^−1^. The cloud points were the temperature at which the appearance of turbidity was observed by optical microscopy (5050zoom, Olympus Co., Tokyo, Japan). DSC (DSC204F1, Netzsch, Selb, Germany) was used for measuring crystallization temperature (T_c_). A small amount of solid mixtures was sealed in an aluminum DSC pan, heated to 180 °C and held for 5 min to erase thermal history and then cooled to room temperature at 10 °C·min^−1^. During the cooling process, the onset of exothermic peak was identified as T_c_.

### 2.3. Fabrication of PVDF-HFP Membrane

First, 30 wt % PVDF-HFP and 70 wt % mixed diluent were fed into a clean and dried glass vessel and stirred at 150 °C for approximately 4 h to obtain a homogeneous solution. After standing for almost 3 h with atmospheric pressure, the bubble in the solution could be removed. The homogeneous solution was cast onto the pre-clean glass substrate with a thickness of 200 μm at 170 °C and then the glass substrate was directly quenched in the water bath (5 °C). The remaining diluent in films was extracted by absolute ethanol which was then removed by immersing in pure water. Finally, the membranes were dried using a drier (FD-1A-50, Shanghai, China). The composition of the obtained PVDF-HFP membranes is exhibited in Table 1.

### 2.4. Characterization

The dried membranes were fractured in liquid nitrogen. The morphologies of membrane surface and cross-section were observed with SEM (TM3030, Hitachi, Tokyo, Japan) after being coated with gold. An atomic force microscope (AFM, Agilent-S5500, Agilent, Palo Alto, CA, USA) was employed to analyze the surface states of the prepared membranes. The hydrophobicity and lipophilicity of membranes were measured by contact angle goniometer (DSA-100, Kruss, Hamburg, Germany) at room temperature. To obtain average value, the contact angle was measured five times at different locations. The mechanical strength tests of obtained membranes were performed with an electron instrument (YG061F Laizhou, Laizhou, China). Each membrane was cut into 5 mm (width) × 30 mm (length) specimen and the tensile rate was 20 mm·min^−1^. Five measurements were performed for every sample. The mean pore size of PVDF-HFP porous membranes was investigated using automatic mercury porosimeter (AutoPore IV-9500, Tektronix, Beaverton, OR, USA) and the porosity of membrane was measured by a gravimetric method and calculated as follows:

(1) ε(%)=(W1−W2)ρA(W1−W2)ρA+W2ρB

where *ε*(%) is the porosity of the membrane; *W_1_* and *W_2_* are dry and wet membrane weights, respectively; and *ρ_A_* and *ρ_B_* are the density of polymer and butanol, respectively.

The enthalpy of fusion and degree of crystallization of obtained membranes were measured using DSC (German Netzsch, DSC204F1, Netzsch, Selb, Germany) and calculated as follows:

(2) $$X_{c}=\frac{\Delta H_{f}}{\Delta H_{f}^{0}}\times 100\%$$

where *X_c_* is the crystallinity of PVDF-HFP membrane, Δ*H_f_* is the fusion enthalpy of PVDF-HFP membrane, and Δ$H_{f}^{0}$ is the fusion enthalpy of PVDF-HFP with 100% crystallinity, i.e., 104.7 J·g^−1^ [24].

### 2.5. Preparation of Surfactant-Free and Surfactant-Stabilized Water-in-Oil Emulsion

To obtain a homogeneous turbid solution, 1 mL pure water was added dropwise into 100 mL diesel and vigorously stirred for at least 6 h. After standing for 1 day, the surfactant-free water-in-oil emulsion was prepared. For surfactant-stabilized water-in-oil emulsion, 0.2 g span80 with a hydrophile–lipophile balance (HLB) value of 4.3 was added into 100 mL diesel and vigorously stirred for 0.5 h. Then, 1 mL pure water was added dropwise into the mixture and stirred vigorously for 6 h to obtain surfactant-stabilized water-in-oil emulsion. After standing for more than 7 days, no obvious agglomeration and precipitation was observed.

### 2.6. Surfactant-Free and Surfactant-Stabilized Emulsion Separation Experiments

The prepared PVDF-HFP membrane was placed on the porous support between feeding bottle and filter tip and sealed carefully by Teflon tape. The separation experiment was carried out at a negative pressure (−0.09 MPa). The filtration rate was measured on the permeated volume of surfactant-free or surfactant-stabilized water-in-oil emulsion through the membrane and determined as follows:

(3) $$J=\frac{V}{S\times t}$$

where *J* is the filtration rate (L·m^−2^·h^−1^), *V* is the permeate quantity (L), *S* is the effective area (m^2^) and *t* is the testing time (h).

The separation efficiency of the surfactant-free or surfactant-stabilized water-in-oil emulsion was calculated as follows:

(4) $$R\left(\%\right)=\frac{C_{f}-C_{p}}{C_{f}}\times 100\%$$

where *C_f_* and *C_p_* are water content of feed and permeate water-in-oil emulsion, respectively; and *R* is the separation efficiency. To get the average value, every membrane was tested at least five times.

The water content in the filtrate was detected by using the Karl Fischer titrator (C20, Mettler Toledo, Greifensee, Switzerland) and then the purity was analyzed to obtain the filter fineness. Photographs of the emulsion and filtrate were taken with a Nikon optical microscope (E200, Nikon, Tokyo, Japan).

### 2.7. Antifouling Performance

To evaluate the antifouling performance of the obtained membrane, the filtration rate of the membrane M3-S2 for treatment of emulsions separation in 15 cycles was performed. After each cycle, the membrane was carefully taken out and simply washed with ethanol, and then dried in air.

## 3. Results and Discussion

### 3.1. Phase Diagram

**Figure 2:**
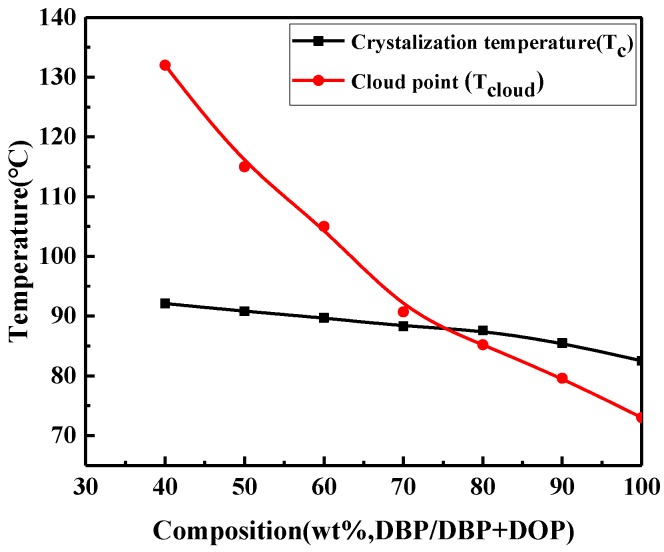
Phase diagram of 30 wt % PVDF-HFP membranes prepared with mixed diluent of different DBP contents.

The phase diagram of polymer/diluent system has important data for preparing membranes via TIPS. The phase diagram for this system (30 wt % PVDF-HFP and 70 wt % mixed diluents with varied DBP/DOP ratios) is shown in Figure 2. In Figure 2, it is seen clearly that T_c_ decreased slowly with the increase of DBP/DOP ratio, while T_cloud_ decreased rapidly. When the DBP/DOP ratio was larger than 7/3, T_c_ was higher than T_cloud_ and the S-L phase separation preceded L-L phase separation. With the further increase of DOP content, the L-L phase separation preceded the S-L phase separation. In addition, the relation between DBP content in mixed diluent was shown in Supplementary Materials.

### 3.2. The Effect of Mixed Diluent Compositions on PVDF-HFP Membranes

**Figure 3:**
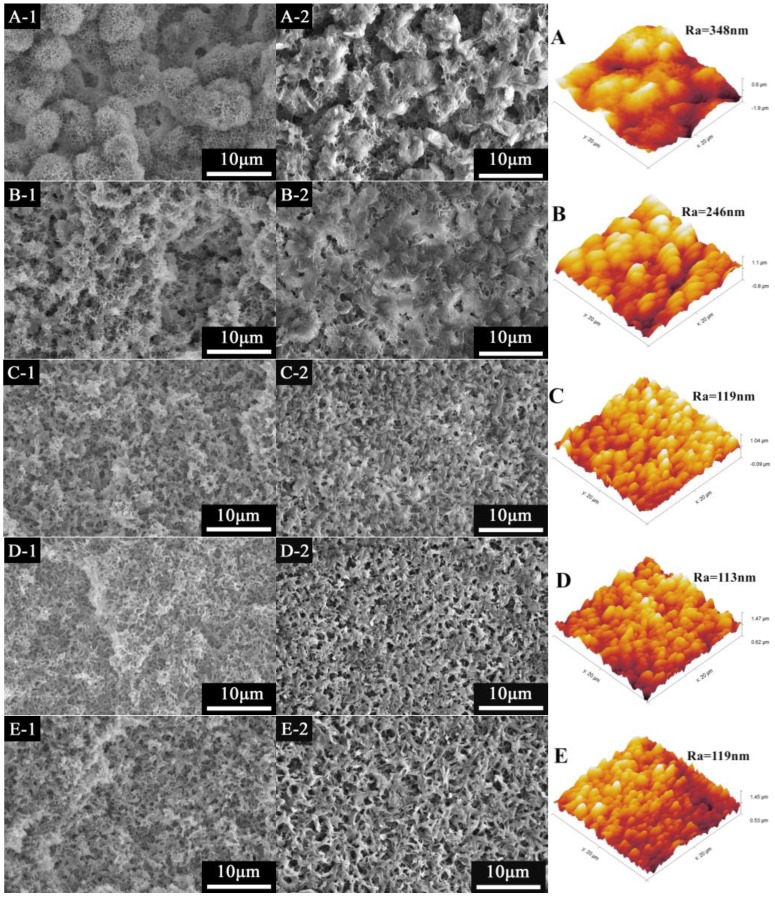
The SEM and AFM morphology of PVDF-HFP membrane: (**A**–**E**) M0–M4 (1: cross section; 2: surface).

During the membrane preparation, the kinetic factors and thermodynamic factors are important for membrane structure [25]. The membrane structure can be controlled by the preparing condition, such as polymer concentration, cooling rate and interaction between polymer and diluent [26]. The ratio of DBP/DOP had an obvious effect on the structure of PVDF-HFP membranes. In Figure 3(A-1), it is seen clearly that the cross section entirely presented spherulitic structure when the diluent was only DBP. The reason for the formation of spherulites depended on the strong interaction between PVDF-HFP and DBP which caused the S-L phase separation [27]. As the DBP/DOP ratio decreased (but larger than 7/3), the spherulite size became smaller and the section structure denser. It can be explained as the higher DBP/DOP ratio gave rise to stronger interaction between PVDF-HFP and mixed diluent. The strong interaction facilitated occurrence of S-L phase separation before L-L phase separation and spherultic structure can be obtained. Additionally, the stronger interaction made the system present stronger nucleation activity and less primary nuclei formed. When the polymer started to crystallize, diluent was excluded from the spherulites easily. The less primary nuclei formed larger spherulites and led to bigger pores. When the ratio decreased further, the structure presented bicontinuous structure gradually. In addition, the bicontinuous structure got much looser with the decrease of DBP/DOP ratio. The reason is that, when the ratio of DBP/DOP was low enough, L-L phase separation can occur before S-L phase separation and the L-L phase separation was driven by the nucleation and growth of the lean-phase. In addition, the lower DBP/DOP ratio made the interaction between PVDF-HFP and mixed diluent get weaker. The weaker interaction led to more time for L-L phase separation, leading to looser structure with larger pores.

From the surface SEM and AFM images of PVDF-HFP membranes, it can be found clearly that the surface of M0 was composed of spherulites and surface roughness was considerably higher than that of other membranes. As the DBP/DOP ratio decreased (but larger than 7/3), the membrane surface became denser due to the smaller space between spherulites. In addition, the surface became smoother with the decrease of DBP/DOP ratio, which was verified by the AFM images. With further increase of DOP content, the surface became more porous and roughness parameters increased.

**Figure 4:**
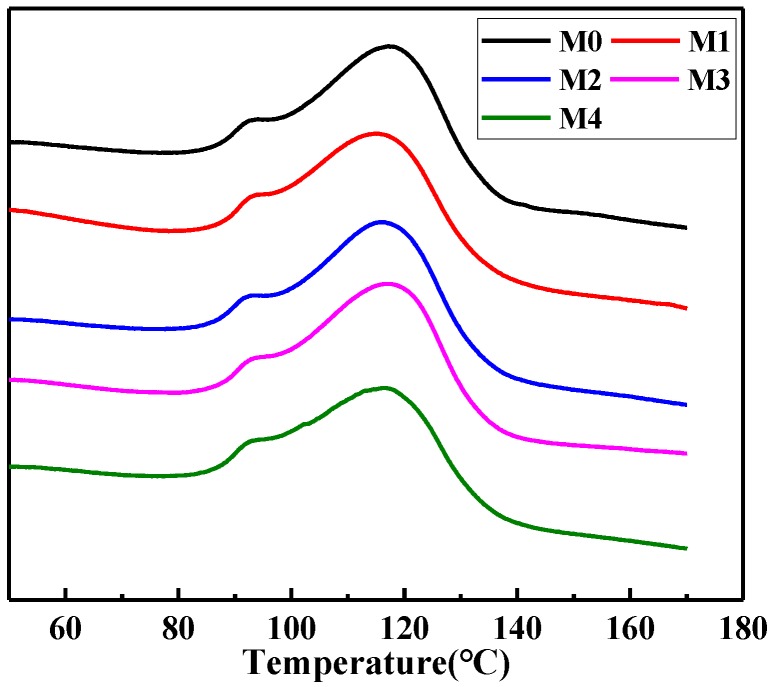
DSC diagram of the melting curves of the PVDF-HFP membranes prepared with mixed diluents.

As discussed above, the ratio of DBP/DOP had an effect on the phase separation process. Therefore, it also influenced the degree of crystallization of PVDF-HFP membrane. The crystallinity was determined by DSC (Figure 4) and the fusion enthalpy and crystallinity are summarized in Table 2. It was noticed clearly that the crystallinity of the obtained membranes decreased with the decrease of DBP/DOP ratio. When the diluent was only DBP, the growth of spherulites gave the high crystallinity during the S-L phase separation process. With the decrease of DBP/DOP ratio, L-L phase separation was observed and led to the formation of polymer-lean phase during L-L phase separation, which facilitated the formation of amorphous phase of the membrane.

**Figure 5:**
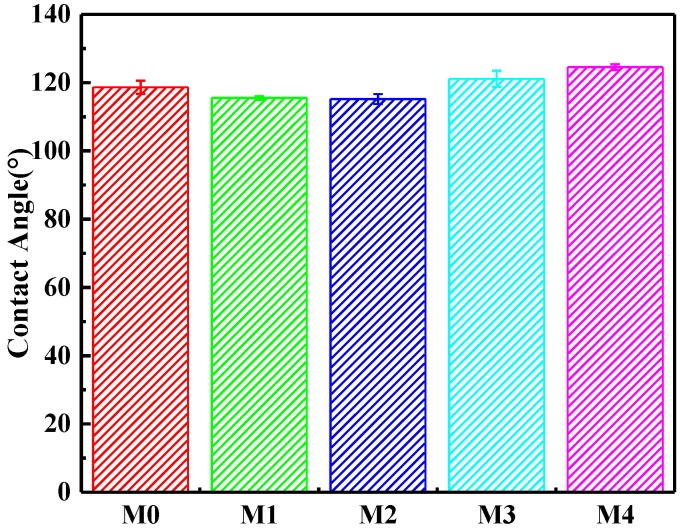
Influence of DBP/DOP ratio on the static water contact angle.

**Figure 6:**
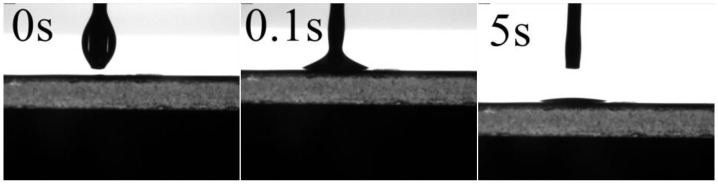
Variation of oil (diesel) contact angle depended on time for upper surface of M3.

Lipophilic-hydrophobic surface of membrane was a key factor for the water in oil emulsion separation effectively [28]. To investigate the selective wettability of the prepared membranes, a water or diesel droplet was used to measure the water or oil contact angle. In Figure 5, it can been seen that the water contact angle decreased firstly with the increase of DOP content. When the DBP/DOP ratio was larger than 7/3, the water contact angle increased with the increase of DOP content. As an example to the PVDF-HFP membrane (M3), as shown in Figure 6, the oil droplet was dropped on the membrane surface and spread out rapidly. At the same time, the oil droplet penetrated the membrane and the oil contact angled decreased to almost 0° within 5 s, which illustrated the excellent oil wettability of PVDF-HFP membranes. In addition, the surface morphology such as micro- and nanostructure had an obvious effect on membrane selective wettability. According to membrane morphology and the results in Figure 5 and Figure 6, it is summarized that the PVDF-HFP membrane had excellent lipophilicity and hydrophobicity.

In consideration of lipophilicity and hydrophobicity of membrane, it was promising to separate surfactant-free water-in-oil emulsion. In the surfactant-free water-in-oil emulsion separation experiments, these membranes can repeatedly separate the immiscible surfactant-free water-in-oil emulsion with filtration rate and separation efficiency, as shown in Table 3. It was reported that the membrane M0 exhibited higher filtration rate (894.41 L·m^−2^·h^−1^) than other membranes. The reason is that surfactant-free water-in-oil emulsion passed through the membrane with large pores easily. With the decrease of DBP/DOP ratio, the filtration rate first decreased and then increased. This changing trend has a direct relation with the membrane structure, which first became denser and then looser with decrease of DBP/DOP ratio. Although the filtration rate of membrane M0 was higher than other membranes, the separation efficiency was just 75.51% owing to its larger pores. With the decrease of DBP/DOP ratio, the surface became denser and then more porous, which resulted in the changing trend of filtration rate. From a general view, the highest water rejection in the membrane M3 was acceptable when considering the better hydrophobicity and more applicable pore structure for surfactant-free water-in-oil emulsion. When high separation efficiency is maintained, higher filtration rates mean a membrane is more applicable for surfactant-free water-in-oil emulsion separation. According to the above results, membrane M3 is more applicable for surfactant-free water-in-oil emulsion separation. In addition, some typical properties of the obtained membranes shown in Supplementary Materials also indicated that membrane M3 is better than others for this application.

### 3.3. Effect of SiO_2_ Content on PVDF-HFP Hybrid Membranes

**Figure 7:**
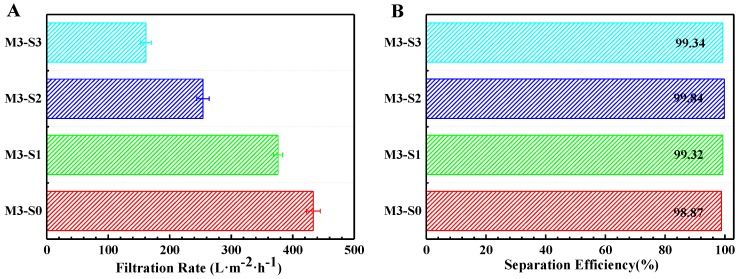
Filtration rate (**A**); and separation efficiency (**B**) for surfactant-stabilized water-in-oil emulsion of the PVDF-HFP hybrid membranes.

**Figure 8:**
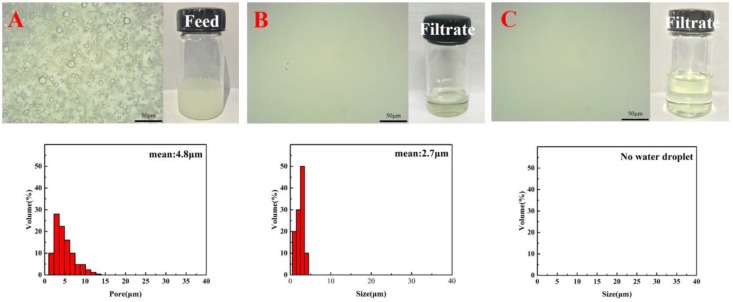
Optical microscope images, photographs and size distribution of surfactant-stabilized water-in-oil emulsion: (**A**) feed; (**B**) M3 filtrate; and (**C**) M3-S2 filtrate.

According to Section 3.2, membrane M3-S0 is more applicable for water-in-oil emulsion separation. Therefore, mixed diluent (DBP/DOP = 7/3) was used to prepare PVDF-HFP hybrid membrane with varying SiO_2_ contents and the structure and properties of obtained hybrid membranes were shown in Supplementary Materials. The filtration rate and separation efficiency for surfactant-stabilized water-in-oil emulsion are presented in Figure 7. The optical microscope images, photographs and size distribution of the emulsion before and after filtrated are shown in Figure 8.

In Figure 7, it can been seen clearly that membrane M3-S0 can separate surfactant-stabilized water-in-oil emulsion with filtration rate of 433.61 L·m^−2^·h^−1^. The introduction of SiO_2_ particles had a significant effect on filtration rate and the filtration rate decreased with the increase of SiO_2_ contents. The reason is that the introduction of SiO_2_ particles facilitated the formation of some micro/nano-protrusions on membrane surfaces, and, with the increase of SiO_2_ contents, more and more microspheres were formed, which blocked the membrane pores. The denser membrane structure suppressed surfactant-stabilized water-in-oil emulsion from passing through the membrane. Compared with the result in Table 3, it was found that the filtration rate for surfactant-stabilized water-in-oil emulsions was lower than that for surfactant-free water-in-oil emulsion because the demulsification process weakened the filtration rate for surfactant-stabilized emulsions significantly.

Although the filtration of membrane M3-S0 was higher than others, the separation efficiency was just 98.87% owing to its looser surface and relatively poor hydrophobicity. With the addition of SiO_2_ particles, the surface became denser, which resulted in lower filtration rate. However, the separation efficiency was larger than 99% owing to its better hydrophobicity and denser structure. The above results are also shown in Figure 8. Firstly, from the photographs of emulsions before and after separation process, it is clearly seen that the emulsion became very clear after being filtrated. Secondly, from the optical microscope images, it is found that there were many emulsion droplets in initial emulsion, whereas there were almost no emulsion droplets in filtrate after being filtrated by the membrane M3-S2.

**Figure 9:**
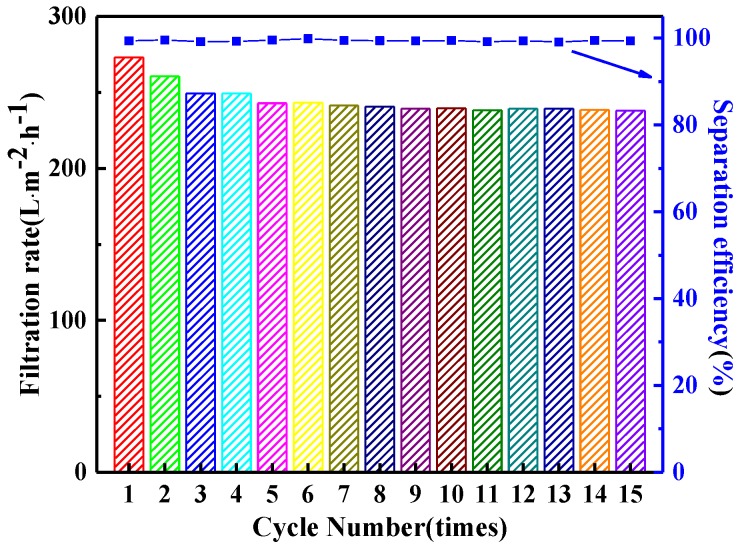
Filtration rate and separation efficiency filtration flux of the membrane M3-S for repeating separation experiment.

During the long running time, unavoidable membrane fouling occurs, which leads to lower filtration rate and separation efficiency. In this work, a repeating separation experiment is applied to evaluate the antifouling performance of the membrane M3-S2. As shown in Figure 9, the change of filtration flux with cycle number is small. In addition, the separation efficiency had no discernable difference during the repeating experiment. These results indicated an excellent antifouling property of the membrane M3-S2. In addition, a comparison of the mechanical property and separation performance for surfactant-stabilized water-in-oil emulsion between the PVDF-HFP hybrid membranes and PVDF membranes reported in the literature has been made, as shown in Table S5. PVDF-HFP membrane possessed better mechanical properties due to its inherent and outstanding flexibility. Furthermore, the excellent hydrophobicity and desirable pore size of PVDF-HFP membrane resulted in higher filtration rate and separation efficiency for surfactant-stabilized water-in-oil emulsion.

## 4. Conclusions

In this paper, novel PVDF-HFP porous membranes are fabricated via TIPS for oil/water separation. The effects of mixed diluent and additives of the structure and properties are studied. The phase diagram of the ternary system shows that the T_cloud_ of the system increases quickly, while T_c_ increases slowly with decrease of DBP/DOP ratio. In addition, the main type of phase separation can change from S-L phase separation to L-L phase separation with decrease of DBP/DOP ratio. Therefore, a bicontinuous structure could be obtained by increasing DOP content. In addition, the crystallization of membranes decreases with the decrease of DBP/DOP ratio. The results also show that the membranes prepared with mixed diluent have excellent mechanical properties and hydrophobicity. When the DBP/DOP ratio is 7/3, the obtained membrane reaches optimal properties. In addition, PVDF-HFP hybrid membranes are successfully fabricated with different SiO_2_ content. The PVDF-HFP hybrid membrane with 2 wt % SiO_2_ particles shows higher separation efficiency of 99.84% for the surfactant-stabilized water-in-oil emulsion separation. More importantly, the hybrid membrane also exhibits excellent antifouling performance.

## Figures and Tables

**Table 1:** The composition of the obtained PVDF-HFP membranes.

Membrane	PVDF-HFP/wt %	DBP/wt %	DOP/wt %	SiO_2_/wt %
M0	30	70	-	-
M1	30	63	7	-
M2	30	56	14	-
M3/M3-S0	30	49	21	-
M4	30	42	38	-
M3-S1	29	49	21	1
M3-S2	28	49	21	2
M3-S3	27	49	21	3

**Table 2:** The enthalpy of fusion and crystallinity of the PVDF-HFP membranes prepared with mixed diluent.

Membrane	Fusion Enthalpy (J·g^−1^)	Crystallinity (%)
M0	29.00	27.70
M1	28.69	27.40
M2	27.80	26.56
M3	27.02	25.81
M4	26.08	24.91

**Table 3:** Filtration rate and separation efficiency for surfactant-free water-in-oil emulsion of PVDF-HFP porous membranes.

Membrane	M0	M1	M2	M3	M4
Filtration rate (L·m^−2^·h^−1^)	894.41 ± 103.33	460.42 ± 25.15	277.55 ± 17.63	497.41 ± 67.27	609.43 ± 41.21
Separation efficiency (%)	75.51 ± 6.72	89.21 ± 0.81	95.45 ± 0.41	99.41 ± 0.12	97.56 ± 0.44

## Supplementary Materials

The following are available online at http://www.mdpi.com/1996-1944/11/3/443/s1, Figure S1. Effect of mixed diluent on σ-γ curve (A) and η-γ curve (B); Figure S2. Relation between DBP content in mixed diluent and the difference of solubility parameter (Δδ) between PVDF-HFP and mixed diluent; Figure S3. The SEM morphology of PVDF-HFP hybrid membranes. A–D: M3-S0~M3-S3, 1: cross section, 2: surface; Table S1. The non-Newtonian index of different polymer solutions; Table S2. Some properties for DBP, DOP, and PVDF-HFP; Table S3. The typical properties of prepared PVDF-HFP membranes; Table S4. Characterization of PVDF-HFP hybrid membranes; Table S5. Comparison of the mechanical property and separation performance for surfactant-stabilized water-in-oil emulsion.

## References

[1] Wypych G. (2012). Handbook of Polymers.

[2] Lalia B.S., Guillen E., Arafat H.A., Hashaikeh R. (2014). Nanocrystalline cellulose reinforced PVDF-HFP membranes for membrane distillation application. Desalination.

[3] Shi L., Wang R., Cao Y.M. (2009). Effect of the rheology of poly(vinylidene fluoride-co hexafluropropylene) (PVDF–HFP) dope solutions on the formation of microporous hollow fibers used as membrane contactors. J. Membr. Sci..

[4] Tian X.Z., Jiang X. (2008). Poly(vinylidene fluoride-co-hexafluoropropene) (PVDF-HFP) membranes for ethyl acetate removal from water. J. Hazard. Mater..

[5] Garcia-Payo M.C., Essalhi M., Khayet M. (2010). Effects of PVDF-HFP concentration on membrane distillation performance and structural morphology of hollow fiber membranes. J. Membr. Sci..

[6] Hemmat A., Ghoreishi S.M., Sabet J.K. (2015). Effect of salt additives on the fabrication of poly(vinylidene fluoride-co-hexafluropropylene) (PVDF-HFP) nanofiber membranes for air Gap membrane distillation. Proc. Mater. Sci..

[7] Feng C.S., Wang R., Shi B., Li G.M., Wu Y.L. (2006). Factors affecting pore structure and performance of poly(vinylidene fluoride-co-hexafluoropropylene) asymmetric porous membrane. J. Membr. Sci..

[8] Garcia-Fernandez L., Garcia-Payo M.C., Khayet M. (2014). Effects of mixed solvents on the structural morphology and membrane distillation performance of PVDF-HFP hollow fiber membranes. J. Membr. Sci..

[9] Stephan A.M., Nahm K.S., Kulandainathan M.A., Ravi G., Wilson J. (2006). Poly(vinylidene fluoride-hexafluoropropylene) (PVDF-HFP) based composite electrolytes for lithium batteries. Eur. Polym. J..

[10] Hwang J., Jeong S.K., Nahm K.S., Stephan A.M. (2007). Electrochemical studies on poly(vinylidene fluoride-hexafluoropropylene) membranes prepared by phase inversion method. Eur. Polym. J..

[11] Li G.C., Zhang P., Zhang H.P., Yang L.C., Wu Y.P. (2008). A porous polymer electrolyte based on P(VDF-HFP) prepared by simple phase separation process. Electrochem. Commun..

[12] Wongchitphimon S., Wang R., Jiraratananon R. (2011). Surface modification of polyvinylidene fluoride-co hexafluoropropylene (PVDF-HFP) hollow fiber membrane for membrane gas absorption. J. Membr. Sci..

[13] Cui Z.Y., Xu Y.Y., Zhu L.P., Wang J.Y., Zhu B.K. (2009). Investigation on PVDF-HFP microporous membranes prepared by TIPS process and their application as polymer electrolytes for lithium ion batteries. Ionics.

[14] Cao J.H., Zhu B.K., Xu Y.Y., Li J., Chen C.X. (2008). Preparation and characterization of PVDF-HFP membrane. J. Mracromol. Sci. A.

[15] Ji G.L., Zhu B.K., Cui Z.Y., Zhang C.F., Xu Y.Y. (2007). PVDF porous matrix with controlled microstructure prepared by TIPS process as polymer electrolyte for lithium ion battery. Polymer.

[16] Li X.F., Xu G.Q., Lu X.L., Xiao C.F. (2008). Effects of mixed diluent compositions on poly(vinylidene fluoride) membrane morphology in a thermally induced phase-separation process. J. Appl. Polym. Sci..

[17] Song Z.Y., Xing M.H., Zhang J., Li B.A., Wang S.C. (2012). Determination of phase diagram of a ternary PVDF/γ-BL/DOP system in TIPS process and its application in preparing hollow fiber membranes for membrane distillation. Sep. Purif. Technol..

[18] Wang L., Huang D.X., Wang X.D., Meng X.R., Lv Y.T., Wang X., Miao R. (2015). Preparation of PVDF membranes via the low-temperature TIPS method with diluent mixtures: The role of coagulation conditions and cooling rate. Desalination.

[19] Sun C.G., Feng X.S. (2017). Enhancing the performance of PVDF membranes by hydrophilic surface modification via amine treatment. Sep. Purif. Technol..

[20] Zhang W.B., Shi Z., Zhang F., Liu X., Jin J., Jiang L. (2013). Superhydrophobic and superoleophilic PVDF membranes for effective separation of water-in-Oil emulsions with high flux. Adv. Mater..

[21] Wang J.Q., Wu Z.Y., Li T.T., Ye J.R., Shen L.Q., She Z., Liu F. (2018). Catalytic PVDF membrane for continuous reduction and separation of p-nitrophenol and methylene blue in emulsified oil solution. Chem. Eng. J..

[22] Hou D.Y., Ding C.L., Li K.L., Lin D.C., Wang D.W., Wang J. (2018). A novel dual-layer composite membrane with underwater-superoleophobic/hydrophobic asymmetric wettability for robust oil-fouling resistance in membrane distillation desalination. Desalination.

[23] Lei W.W., Li H., Shi L.Y., Diao Y.F., Zhang Y.L., Ran R., Ni W. (2017). Achieving enhanced hydrophobicity of graphene membranes by covalent modification with polydimethylsiloxane. Appl. Surf. Sci..

[24] Cao J.H., Zhu B.K., Xu Y.Y. (2006). Structure and ionic conductivity of porous polymer electrolytes based on PVDF-HFP copolymer membranes. J. Membr. Sci..

[25] Van de Witte P., Dijkstra P.J., Van den Berg J.W.A., Feijen J. (1996). Phase behavior of polylactides in solvent-nonsolvent mixtures. J. Polym. Sci. Pol. Phys..

[26] Ji G.L., Zhu L.P., Zhu B.K., Zhang C.F., Xu Y.Y. (2008). Structure formation and characterization of PVDF hollow fiber membrane prepared via TIPS with diluent mixture. J. Membr. Sci..

[27] Kim J.F., Jung J.T., Wang H.H., Lee S.Y., Moore T., Sanguineti A., Drioli E., Lee Y.M. (2016). Microporous PVDF membranes via thermally induce phase separation (TIPS) and stretching methods. J. Membr. Sci..

[28] Lee C.H., Johnson N., Drelich J., Yap Y.K. (2011). The performance of superhydrophobic and superoleophilic carbon nanotube meshes in water-oil filtration. Carbon.

