# Supplementary material for: Poly(vinylidene Fluoride-Hexafluoropropylene) Porous Membrane with Controllable Structure and Applications in Efficient Oil/Water Separation
Source: Materials (Basel). 2018 Mar 18;11(3):443. doi: 10.3390/ma11030443 (PMC5873022; doi:10.3390/ma11030443)
Supplement: Supplementary file 1 [file materials-11-00443-s001.pdf]

materials

MDPI

Supplementary

Poly(vinylidene Fluoride-Hexafluoropropylene) Porous Membrane with Controllable Structure and Applications in Efficient Oil/Water Separation

Xinya Wang, Changfa Xiao *, Hailiang Liu, Qinglin Huang, Junqiang Hao and Hao Fu

State Key Laboratory of Separation Membranes and Membrane Processes, National Center for International Joint Research on Separation Membranes, Tianjin Polytechnic University, No. 399, Binshui Road, Xiqing District, Tianjin 300387, China; wangxy0914@163.com (X.W.); liuhailiang723@163.com (H.L.); huangqinglin@tjpu.edu.cn (Q.H.); 15222696965@163.com (J.H.); fuhao0823@163.com (H.F.)

* Correspondence: cfxiao@tjpu.edu.cn; Tel: +86-022-83955299

Received: 1 February 2018; Accepted: 18 March 2018; Published: 18 March 2018

1. Rheological Properties of Polymer Solutions

The molecular state and gelation behavior of polymer solutions can be obtained from the rheological measurements [1,2]. In addition, the rheological properties of polymer solutions have obvious effect on its processibility [3]. The effect of ratio of DBP/DOP on the rheological behavior of the polymer solutions is presented in Figure S1. The shear stress (σ) of the PVDF-HFP/DBP solutions was found to be higher than that of the PVDF-HFP/DBP/DOP solutions at the same shear rate (γ). In addition, the viscosity (η) of polymer solutions decreased with the addition of DOP content. The shear stress and viscosity continuously decreased with an increase in the content of DOP. As to further investigate the effects of mixed diluent on the non-Newtonian behavior of polymer solutions, the non-Newtonian index (n), slope of the curves of logarithm of shear stress versus logarithm of shear rate, was presented in Table S1. The value of the non-Newtonian index (n) had no obvious difference with addition of DOP and the non-Newtonian indexes of all polymer solutions were close to 1. Taken together, the polymer solutions exhibited excellent Newtonian flowing property.

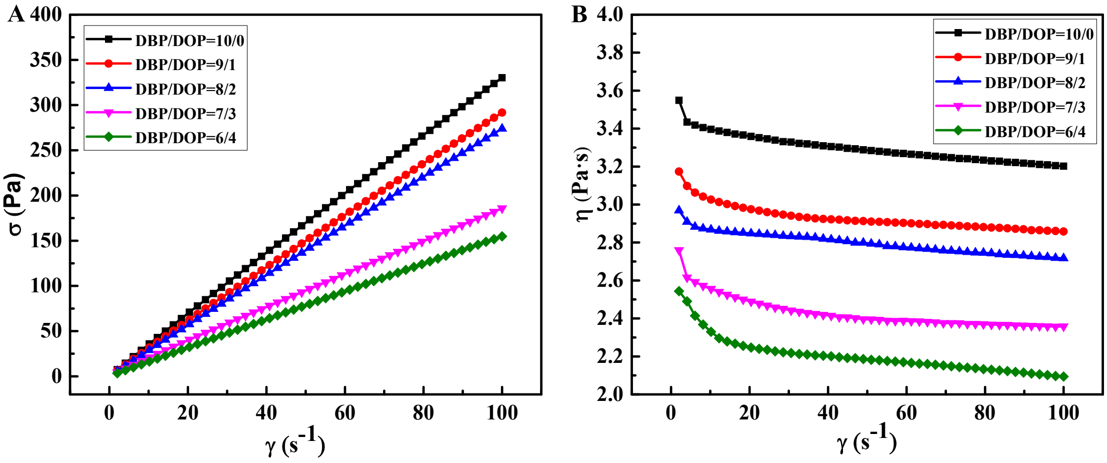

Figure S1. Effect of mixed diluent on σ-γ curve (A) and η-γ curve (B).

Table S1. The non-Newtonian index of different polymer solutions.

Temperature(°C)	Polymer Solution	M0	M1	M2	M3	M4
150	Non-Newtonian index (n)	0.9768	0.9722	0.9870	0.9776	0.9821

2. Relation between DBP Content in Mixed Diluent

The relation between DBP content in mixed diluent and the difference of solubility parameter (Δδ) between PVDF-HFP and mixed diluent was shown in Figure S2. The solubility parameter of mixed diluent and the difference of solubility parameter between PVDF-HFP and mixed diluent were calculated by the following equation:

$$\delta_i = \delta_1\phi_1 + \delta_2\phi_2 \quad (1)$$

$$\Delta\delta = |\delta_i - \delta_j| \quad (2)$$

where δ1 and δ2 were solubility parameter of one diluent and another one (exhibited in Table S2), δi and δj were solubility parameter of mixed diluent and PVDF-HFP. It was clear that Δδ was decreasing proportionally with the increase of the DBP content, which expressed that the increase of the DBP content enhanced the interaction between the polymer and the mixed diluent. Furthermore, DBP provided higher viscosity than DOP. The effect of the stronger interaction and higher viscosity on membrane structure was discussed detailed in section 3.2.

Table S2. Some properties for DBP, DOP, and PVDF-HFP.

Substance	Density (g·cm^{-3})	Solubility parameter [(MPa)$^{1/2}$]	Molecular weight	Viscosity [mpa.s (20 °C)]
DBP	1.045	20.3[a]	278.34	163
DOP	0.985	18.2[a]	390.55	80
PVDF-HFP	1.770	23.2[5]	-	-

[a]Ref. [4]

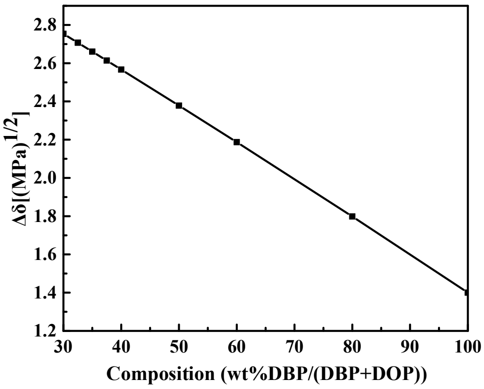

Figure S2. Relation between DBP content in mixed diluent and the difference of solubility parameter (Δδ) between PVDF-HFP and mixed diluent.

3. Typical Properties of PVDF-HFP Membranes

Some typical properties of the obtained membranes were tested and listed in Table S3. It can be clearly seen that the mean pore size and porosity of obtained membranes decreased firstly and then increased with the decrease of DBP/DOP ratio. When DBP/DOP ratio was larger than 7/3, the main type of phase separation was L-L phase separation and the size of spherulite and the space between spherulites decreased with the DBP/DOP ratio in mixed diluent. Hence, the mean pore size and porosity decreased. As the DBP/DOP ratio decreased further, the main phase separation was L-L phase separation and the bicontinuous structure formed during the cooling process. Therefore, the mean pore size and porosity increased.

It was well known that the mechanical properties were mainly depended on the crystallinity and membrane structure. When the DBP/DOP ratio was larger than 7/3, both the breaking strength and breaking elongation decreased gradually with the increase of DOP content due to the formation of bigger space between spherulites and lower crystallinity. When DBP/DOP ratio was 7/3, the breaking strength and breaking elongation was largest. Moreover, with the further increase of DOP content, the bicontinuous structure got much looser which resulted in worse mechanical properties.

Table S3. The typical properties of prepared PVDF-HFP membranes.

Membrane	Mean Pore Size (nm)	Porosity (%)	Breaking Strength (MPa)	Breaking Elongation (%)
M0	939.3±0.01	53.8±5.3	2.07±0.09	246.5±19.10
M1	827.1±0.03	47.4±4.1	2.68±0.05	372.4±14.01
M2	734.6±0.03	45.3±6.4	2.88±0.09	1053.4±28.64
M3	627.1±0.02	53.8±3.1	3.27±0.08	1132.6±19.80
M4	994.1±0.01	54.6±4.7	2.07±0.14	714.6±13.87

4. Effect of SiO_2 Content on PVDF-HFP Hybrid Membranes

From the SEM images of cross section, it was found that spherulites increased with the increase of SiO2 contents. The reason was that the SiO2 particles as the primary nuclei promoted the S-L phase separation and the spherulitic structure instead of bicontinous structure. It can been also seen clearly that the mixed diluent facilitated the formation of membranes with a rough and porous surface. Then with the addition of SiO_2 particles, some micro/nano-protrusions were scattered on the membrane surfaces. With the increase of SiO_2 contents, microsphere was formed and blocked the membrane pore size which resulted in the lower porosity and smaller pores. In addition, the introduction of SiO_2 particles facilitated the formation of rougher membrane surface. Furthermore, the formation of micro/nano-protrusions made the hybrid membrane more hydrophobic which can be testified by higher water contact angle (Table S4).

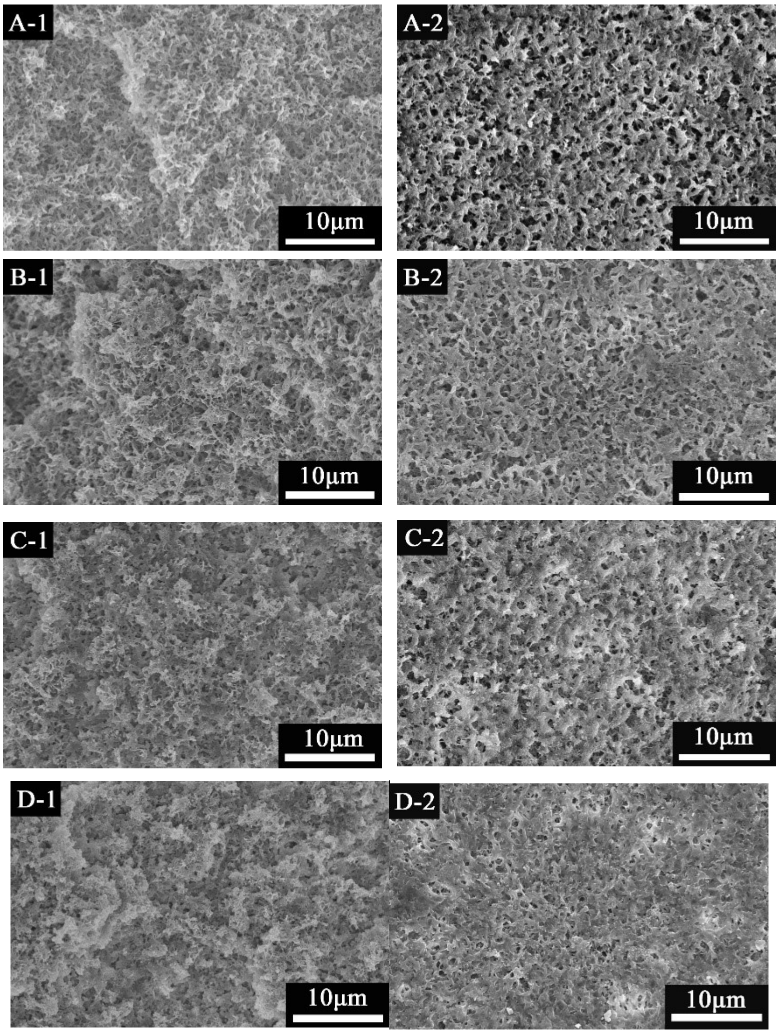

Figure S3. The SEM morphology of PVDF-HFP hybrid membranes. **A**–**D**:M3-S0~M3-S3,1:cross section, 2:surface.

Some typical properties of PVDF-HFP hybrid membranes were presented in Table S4. It was found that the mean pore size and porosity of PVDF-HFP hybrid membranes decreased which has been explained in last section. In addition, the hydrophobicity of hybrid membranes enhanced with the increase in SiO_2 content owing to rougher membrane surface.

With the addition of SiO_2 particles, the breaking strength increased and then decreased. It can be explained that the SiO_2 particles were evenly distributed in the membrane and closely combined with the molecular chains of matrix by physic and chemistry effect because of their small size and surface effect. The region of stress concentration achieved good dispersion and the entanglement point inhibited the movement of molecular chains when the external force was applied. However, an excessive amount of SiO_2 particles could cause the agglomeration which resulted in the decrease of breaking strength. On the contrary, the breaking elongation declined with the addition of SiO_2 particles. The reason was that the low surface energy of fluoropolymer had the low surface energy which resulted in poor affinity between fluoropolymer with inorganic particles [6]. PVDF-HFP and SiO_2 particles used in this work were also true for this theory.

Table S4. Characterization of PVDF-HFP hybrid membranes.

Membrane	Mean Pore size (nm)	Porosity (%)	Contact Angle (°)	Breaking Strength (MPa)	Breaking Elongation (%)
M3	734.6	54.6±3.1	127.98±2.25	3.27±0.08	1132.6±19.80
M3-S1	643.0	51.3±1.4	131.47±3.12	3.49±0.17	771.1±22.74
M3-S2	552.5	47.6±2.7	135.02±1.67	3.92±0.31	540.43±13.46
M3-S3	374.7	43.2±2.6	135.72±1.47	3.29±0.24	338.67 ±17.17

Table S5. Comparison of the mechanical property and separation performance for surfactant-stabilized water-in-oil emulsion.

Reference	Membrane	Breaking Strength (MPa)	Breaking Elongation (%)	Filtration Rate ($L \cdot m^{-2} \cdot h^{-1}$)	Separation Efficiency (%)
This work	M3-S0	3.27 ± 0.08	1132.6 ± 9.80	433.61 ± 11.27	98.87
	M3-S2	3.92 ± 0.31	540.43 ± 13.46	254.17 ± 10.14	99.84
[7]	PVDF membrane	-	-	200-300	99.64–99.80
[8]	PVDF modified membrane	2	<30	≈700	99.95
[9]	PVDF/SA nanofibers	-	-	≈200	<99.5
[10]	PVDF/PMMA composite membrane	-	-	≈250	≈99.5
[11]	PVDF membrane	-	-	220-3000	99.02-99.98

References

1. Huang, Q.L.; Huang Y.; Xiao C.F.; You Y.W.; Zhang C.X. Electrospun ultrafine fibrous PTFE-supported ZnO porous membrane with self-cleaning function for vacuum membrane distillation. *J. Membr. Sci.* **2017**, *534*, 73–82, doi:10.1016/j.memsci.2017.04.015.
2. Cho, H.J.; Ki, C.S.; Oh, H.; Lee, K.H.; Um, I.C. Molecular weight distribution and solution properties of silk fibroins with different dissolution conditions. Int. *J. Bio. Macromol.* **2012**, *51*, 336–341, doi:10.1016/j.ijbiomac.2012.06.007.
3. Kim, H.J.; Um, I.C. Relationship between rheology and electro-spinning performance of regenerated silk fibroin prepared using different degumming methods. Korea-Aust. *Rheol. J.* **2014**, *26*, 119–125, doi:10.1007/s13367-014-0012-6.
4. Brandrup, J.; Immergut, E.H.; Grulke, E.A. *Polymer Handbook*, 4th ed.; John Wiley & Sons: New York, USA, 1999.
5. Wongchitphimon, S.; Wang, R.; Jiraratananon, R.; Shi, L.; Loh, C.H. Effect of polyethylene glycol (PEG) as an additive on the fabrication of polyvinylidene fluoride-co-hexafluropropylene (PVDF-HFP) asymmetric microporous hollow fiber membranes. *J. Membr. Sci.* **2010**, *369*, 329–338, doi:10.1016/j.memsci.2010.12.008.
6. Liu C.; Chen L.; Zhu L. Fouling behavior of lysozyme on different membrane surfaces during the MD operation: An especial interest in the interaction energy evaluation. *Water Res.* **2017**, *119*, 33–46, doi:10.1016/j.watres.2017.04.041.
7. Chen L.W.; Si Y.F.; Zhu H.I.; Jiang T.; Guo Z.G. A study on the fabrication of porous PVDF membranes by in-situ elimination and their applications in separating oil/water mixtures and nano-emulsions. *J. Membr. Sci.* **2016**, *520*, 760-768, doi:10.1016/j.memsci.2016.08.026.
8. Zhang W.B.; Shi Z.; Zhang F.; Liu X.; Jin J.; Jiang L. Superhydrophobic and superoleophilic PVDF membranes for effective separation of water-in-Oil emulsions with high flux. *Adv. Mater.* **2013**, *25*, 2071–2076, doi:10.1002/adma.201204520.
9. Li J.; Xu C.C.; Tian H.F.; Zha F.; Qi W.; Wang Q. Blend-electrospun poly(vinylidene fluoride)/stearic acid membranes for efficient separation of water-in-oil emulsions. *Colloid. Surf. A* **2018**, *538*, 494–499; doi:10.1016/j.colsurfa.2017.11.043.
10. Yuan X.Y.; Li W.; Zhu Z.G.; Han N.; Zhang X.X. Thermo-responsive PVDF/PSMA composite membranes with micro/nanoscale hierarchical structures for oil/water emulsion separation. *Colloid. Surf. A* **2017**, *516*, 305–316; doi:10.1016/j.colsurfa.2016.12.047.
11. Venault A.; Jumao-As-Leyba A.J.; Chou F.C.; Bouyer D.; Lin I.J.; Wei T.C.; Chang Y. Design of near-superhydrophobic/superoleophilic PVDF and PP membranes for the gravity-driven breaking of water-in-oil emulsions. *J. Taiwan. Inst. Chem. E* **2016**, *65*, 459–471; doi:10.1016/j.jtice.2016.05.011.

© 2018 by the authors. Submitted for possible open access publication under the terms and conditions of the Creative Commons Attribution (CC BY) license (http://creativecommons.org/licenses/by/4.0/).
